# The Gut-Joint Axis in Osteoarthritis

**DOI:** 10.7759/cureus.48951

**Published:** 2023-11-17

**Authors:** Madhan Jeyaraman, Pothuri Rishi Ram, Naveen Jeyaraman, Sankalp Yadav

**Affiliations:** 1 Orthopaedics, ACS Medical College and Hospital, Dr. MGR Educational and Research Institute, Chennai, IND; 2 Orthopaedics and Trauma, Sanjay Gandhi Institute of Trauma and Orthopaedics, Bengaluru, IND; 3 Medicine, Shri Madan Lal Khurana Chest Clinic, New Delhi, IND

**Keywords:** gut, osteoarthritis, gut microbiome, mycobiome, virome

## Abstract

Osteoarthritis (OA) is a complex and prevalent joint disease with a multifaceted pathogenesis, presenting a persistent challenge to medical researchers. However, recent investigations into the gut microbiota (GMB) have unveiled an intriguing connection with OA, giving rise to the concept of the "gut-joint axis". The human gut houses a diverse microbial ecosystem that plays pivotal roles in nutrient synthesis, metabolism, and immune modulation. Dysbiosis, or disruption of this microbial balance, can lead to inflammation through the release of proinflammatory cytokines and the production of inflammatory metabolites. This editorial delves into the evolving understanding of how the GMB may influence OA development and progression. Notably, short-chain fatty acids (SCFAs) produced by gut microorganisms have emerged as potential players in maintaining bone homeostasis and reducing inflammation. Furthermore, compromised gut integrity can lead to endotoxemia and a pro-inflammatory state, contributing to OA. Recent research has highlighted differences in GMB composition and functionality between OA patients and healthy individuals, shedding light on specific microbial taxa and functional pathways associated with OA. The gut mycobiome (fungi) and virome (viruses) in OA remain largely unexplored, presenting exciting opportunities for future investigations. The emerging understanding of the gut-joint axis offers promising avenues for innovative OA prevention and treatment strategies, though further research is needed to fully elucidate these complex interactions.

## Editorial

Osteoarthritis (OA), a chronic joint disease that affects millions worldwide, has long been a puzzle for medical researchers. Its complex pathogenesis, involving inflammation, cartilage degradation, and bone changes, has defied a straightforward explanation. However, a new frontier in OA research is emerging, one that explores the intricate connections between the gut microbiota (GMB) and joint health. In this editorial, we delve into the evolving understanding of how microbial diversity and functionality may influence the development and progression of OA, shedding light on the promising concept of the "gut-joint axis" [1,2].

Human GMB: a diverse ecosystem

The human gut is home to a thriving ecosystem of different microbial species, including bacteria, fungi, viruses, phages, parasites, and archaea. This complex community plays a vital role in synthesizing nutrients, metabolizing substances, and influencing the immune system's function. When this microbial balance, known as eubiosis, is disrupted, it can lead to inflammation by releasing proinflammatory cytokines and producing inflammatory metabolites by gut bacteria [3].

Eubiosis and dysbiosis are terms used to describe the states of the microbial community within the human gut, which consists of trillions of microorganisms, including bacteria, viruses, fungi, and other microorganisms. This complex community is collectively known as the gut microbiota, and it plays a crucial role in maintaining human health.

Eubiosis

Eubiosis refers to a state of balanced and harmonious microbial communities within the gut. In this state, the diversity and abundance of beneficial or commensal microorganisms are in proper balance, and they perform various functions that contribute to human well-being. Some key functions of the gut microbiota in eubiosis include:

Nutrient synthesis: The microbiota helps in synthesizing essential nutrients, such as certain vitamins (e.g., vitamin K and some B vitamins) and short-chain fatty acids (SCFAs), which are important for gut health.

Metabolism: It assists in breaking down complex substances, aiding in the digestion and absorption of food.

Immune regulation: The gut microbiota plays a role in educating the immune system, helping it to distinguish between harmful and harmless substances and thereby influencing immune function.

Dysbiosis

Dysbiosis, on the other hand, refers to an imbalance or disruption in the gut microbiota. It can manifest in various ways, including a reduction in beneficial microbes and an overgrowth of harmful or pathogenic species. Dysbiosis can result from a variety of factors such as:

Diet: Consuming a diet high in processed foods and sugars and low in fiber can promote the growth of harmful microbes.

Antibiotics: These medications can alter the composition of the gut microbiota, potentially reducing beneficial bacteria.

Stress: Chronic stress can impact the gut-brain axis and influence the gut microbiota.

Infections: Certain infections can disrupt the gut microbiota.

Lifestyle factors: Smoking and excessive alcohol consumption can contribute to dysbiosis.

Age and genetics: These factors can also influence the gut microbiota composition.

Consequences of Dysbiosis

When dysbiosis occurs and the microbial balance is disrupted, it can have various negative consequences for human health, including:

Inflammation: Dysbiosis can lead to the release of proinflammatory cytokines and the production of inflammatory metabolites by gut bacteria. This chronic inflammation is associated with a range of health problems, including inflammatory bowel diseases (IBD), obesity, and autoimmune diseases.

Gut disorders: Dysbiosis is a common factor in gut disorders such as irritable bowel syndrome (IBS) and Crohn's disease.

Metabolic disorders: An imbalanced gut microbiota can contribute to metabolic disorders like obesity and type 2 diabetes.

Weakened immune function: Dysbiosis can negatively impact the immune system, potentially making individuals more susceptible to infections and autoimmune diseases.

Gut-joint axis: connecting the dots

Recent research has unveiled a profound connection between GMB and OA. Several factors linked to OA, such as aging, obesity, diet, exercise, joint abnormalities, and trauma, are closely intertwined with GMB. This intricate relationship has given rise to the concept of the "gut-joint axis," suggesting that GMB may hold the key to understanding OA's pathogenesis [4].

Microbial metabolites and OA

Among the many microbial communities, short-chain fatty acids (SCFAs) have emerged as potential players in OA. SCFAs, produced by gut microorganisms, are believed to maintain bone homeostasis, reduce inflammation, and inhibit bone resorption by directly affecting osteoclast activity [5]. Moreover, a compromised intestinal barrier, often referred to as "leaky gut syndrome," can lead to the translocation of microbial products into the bloodstream, causing endotoxemia and a pro-inflammatory state. In animal studies, transferring fecal microbiota from donors who had metabolic problems sped up the development of OA, demonstrating a clear connection between the gut microbiota and the onset of OA [6].

Viruses and OA: a new frontier

Research into the role of the gut bacteriome in OA has been advancing, but the investigation of viruses in joint diseases has been somewhat restricted. While studies have looked at human endogenous retrovirus W and the Epstein-Barr virus in relation to OA, the potential involvement of the mycobiome (fungi) and virome (viruses) in OA remains largely unexplored. This highlights a significant knowledge gap in our understanding of the connection between the gut and joints and emphasizes the necessity for more in-depth research in this area.

Unraveling GMB in OA patients

Recent research has provided intriguing insights into OA patients' GMB composition and functionality. Whole-metagenome shotgun sequencing of fecal samples from OA patients and age- and body mass index (BMI)-matched healthy individuals has revealed notable differences [6].

Biodiversity and phylogenetic composition

While the observed species in the gut bacteriome of OA patients were slightly more abundant than in healthy controls, measures of biodiversity, such as the Shannon diversity index and Simpson index, were similar between the two groups [7].

The Shannon diversity index is a measure of the microbial diversity within a community. It takes into account both the number of different species (species richness) and the distribution of those species (species evenness). In other words, it assesses not only how many species are present but also how evenly they are distributed in the community. Higher values of the Shannon diversity index indicate a more diverse microbial community.

The Simpson index is another measure of microbial diversity, but it focuses on species dominance within a community. It calculates the probability that two randomly selected individuals in a community belong to the same species. In other words, it quantifies the dominance of a few species in the community. Lower values of the Simpson index suggest higher diversity, as it indicates that no single species dominates the community.

However, principal-coordinate analysis (PCoA) indicated a significant distinction in the overall gut microbial structure between OA patients and healthy individuals. Specifically, the gut bacteriome of OA patients exhibited higher levels of Actinobacteriota and Proteobacteriaand lower levels of *Firmicutes* compared to healthy controls. At the genus and species levels, several differences emerged. OA patients displayed enrichment of genera such as *Anaerostipes*, *Bifidobacterium*, *Brachyspira*, and *Eggerthella*, while healthy controls had higher levels of genera like *Faecalibacterium*, *Lachnoclostridium*, *Phascolarctobacterium*, and *Paraprevotella* [7]. Notably, 279 bacterial species exhibited significant differences in relative abundance between the two cohorts, with 41 species enriched in OA patients and 238 in healthy controls.

These differences could have several implications for OA which are as follows:

Inflammatory impact: The variations in bacterial species abundance may impact the inflammatory response in OA. Certain species enriched in OA patients might contribute to inflammation, a hallmark of the condition while others in healthy controls could have anti-inflammatory properties.

Metabolism and diet: These differences may also affect how individuals process nutrients, which is relevant to OA. Some bacterial species are involved in fermenting dietary fibers, producing SCFAs with known anti-inflammatory properties.

Microbial dysbiosis: The observed variations might indicate microbial dysbiosis, an imbalance linked to various health conditions. This dysbiosis could potentially play a role in the development or progression of OA.

Treatment implications: Understanding the specific bacterial species associated with OA may inform potential treatment strategies. Targeting or modulating these species could be explored as interventions to manage or mitigate the condition.

Functional composition of the gut bacteriome

Functional analysis of the gut bacteriome revealed that the prokaryotic functional compositions of OA patients and healthy subjects were similar in terms of biodiversity. However, when utilizing Principal Coordinate Analysis (PCoA), notable differences were observed in the functional composition between the two groups. Specifically, 15 distinct pathways exhibited significant differences in their composition and activity. Some pathways, such as a partial tricarboxylic acid (TCA) cycle and inosine-59-phosphate biosynthesis III, were enriched in OA patients, while others, like chorismate biosynthesis and purine ribonucleosides degradation, were more abundant in healthy controls [7]. The differential enrichment of specific pathways, such as the tricarboxylic acid cycle and inosine-59-phosphate biosynthesis III in OA patients, along with the depletion of pathways like chorismate biosynthesis and purine ribonucleosides degradation in healthy controls, implies a complex interplay of metabolic, immune response, and dietary factors in OA pathogenesis, offering prospects for identifying disease biomarkers and developing precision therapeutic interventions.

Gut mycobiome in OA

Exploring the gut mycobiome (fungi) in OA patients revealed interesting findings. While within-sample biodiversity analysis indicated no significant differences in fungal composition between OA and healthy groups, PCoA demonstrated a notable distinction. At the genus level, OA patients had higher abundances of *Saccharomyces*, *Cryptococcus*, and *Candida* while healthy controls exhibited a dominance of *Saccharomyces*, *Cryptococcus*, and *Malassezia*. *Malassezia* and *Candida *showed significant differences in abundance between the two cohorts [6].

The differences in the gut mycobiome (fungal composition) between OA patients and healthy controls could have several potential consequences:

Immunomodulation: Fungal species can interact with the immune system. The differences in Saccharomyces, Cryptococcus, Candida, and Malassezia abundance can influence the immune response in OA. These variations could potentially exacerbate inflammation or contribute to immune dysregulation, which is a known factor in OA.

Diet and metabolism: Fungal species in the gut can impact dietary metabolism and nutrient absorption. Differences in these fungi may be linked to variations in dietary habits between the two groups, and these dietary variations could, in turn, play a role in OA development or progression.

Microbial interactions: Fungal species can interact with bacteria in the gut. Variations in fungal composition may influence the balance and interactions between different microbial groups. This could have downstream effects on the gut microbiome's overall structure and function, potentially affecting metabolic pathways or inflammatory responses.

Disease biomarkers: Differences in fungal composition could serve as potential biomarkers for OA. The identification of specific fungi that significantly differ between OA patients and healthy controls may offer diagnostic or prognostic insights.

Future directions

The emerging understanding of the gut-joint axis and its implications for OA is a promising avenue of research. However, many questions remain unanswered. Comprehensive investigations into the gut bacteriome, mycobiome, and virome in OA patients are needed to fully elucidate their roles in disease development and progression. Understanding these complex interactions may open doors to innovative prevention and treatment strategies for OA.

The connection between GMB and OA represents a fascinating and evolving field of study. The gut-joint axis concept highlights the intricate interplay between gut microorganisms and joint health. As research in this area progresses, we may uncover novel therapeutic avenues for managing this prevalent and debilitating condition. While there is much more to learn, the potential impact on the lives of OA patients is both promising and exciting.
